# Physical Exercise Exacerbates Acute Kidney Injury Induced by LPS via Toll-Like Receptor 4

**DOI:** 10.3389/fphys.2020.00768

**Published:** 2020-07-17

**Authors:** Talita Guerreiro Rodrigues Húngaro, Leandro Ceotto Freitas-Lima, Marcos Fernandes Gregnani, Mauro Sérgio Perilhão, Thaís Alves-Silva, Adriano Cleis Arruda, Jonatan Barrera-Chimal, Gabriel Rufino Estrela, Ronaldo Carvalho Araújo

**Affiliations:** ^1^Laboratório de Genética e Metabolismo do Exercício, Programa de Nefrologia, Departamento de Biofísica, Universidade Federal de São Paulo, São Paulo, Brazil; ^2^Laboratório de Genética e Metabolismo do Exercício, Programa de Biologia Molecular, Departamento de Biofísica, Universidade Federal de São Paulo, São Paulo, Brazil; ^3^Instituto de Investigaciones Biomédicas, Universidad Nacional Autónoma de México, Mexico City, Mexico; ^4^Unidad de Investigación UNAM-INC, Instituto Nacional de Cardiología Ignacio Chávez, Mexico City, Mexico; ^5^Departamento de Oncologia Clínica e Experimental, Disciplina de Hematologia e Hematoterapia, Universidade Federal de São Paulo, São Paulo, Brazil

**Keywords:** physical exercise, inflammation, TLR-4, kidney, LPS, acute kidney injury, AOAH, lipopolysaccharide

## Abstract

**Introduction:** Lipopolysaccharide (LPS) is a systemic response-triggering endotoxin, which has the kidney as one of its first targets, thus causing acute injuries to this organ. Physical exercise is capable of promoting physiological alterations and modulating inflammatory responses in the infectious process through multiple parameters, including the toll-like receptor (TLR)-4 pathway, which is the main LPS signaling in sepsis. Additionally, previous studies have shown that physical exercise can be both a protector factor and an aggravating factor for some kidney diseases. This study aims at analyzing whether physical exercise before the induction of LPS endotoxemia can protect kidneys from acute kidney injury.

**Methods:** C57BL/6J male mice, 12 weeks old, were distributed into four groups: (1) sedentary (control, *N* = 7); (2) sedentary + LPS (*N* = 7); (3) trained (*N* = 7); and (4) trained + LPS (*N* = 7). In the training groups, the animals exercised 5×/week in a treadmill, 60 min/day, for 4 weeks (60% of max. velocity). Sepsis was induced in the training group by the application of a single dose of LPS (5 mg/kg i.p.). Sedentary animals received LPS on the same day, and the non-LPS groups received a saline solution instead. All animals were euthanized 24 h after the administration of LPS or saline.

**Results:** The groups receiving LPS presented a significant increase in serum urea (*p* < 0.0001) and creatinine (*p* < 0.001) concentration and renal gene expression of inflammatory markers, such as tumor necrosis factor alpha and interleukin-6, as well as TLRs. In addition, LPS promoted a decrease in reduced glutathione. Compared to the sedentary + LPS group, trained + LPS showed overexpression of a gene related to kidney injury (NGAL, *p* < 0.01) and the protein levels of LPS receptor TLR-4 (*p* < 0.01). Trained + LPS animals showed an expansion of the tubulointerstitial space in the kidney (*p* < 0.05) and a decrease in the gene expression of hepatic AOAH (*p* < 0.01), an enzyme involved in LPS clearance.

**Conclusion:** In contrast to our hypothesis, training was unable to mitigate the renal inflammatory response caused by LPS. On the contrary, it seems to enhance injury by accentuating endotoxin-induced TLR-4 signaling. This effect could be partly due to the modulation of a hepatic enzyme that detoxifies LPS.

## Introduction

Physical exercise is able to prevent diseases, to increase our ability to deal with pro-inflammatory patterns, and to improve health in several pathological affections (Hung et al., 2013; El-Kader et al., 2014; Wegner et al., 2014; Sharman et al., 2015). It is well established that physical exercise has the ability to reduce inflammatory responses, tissue damage, and mortality in cases of sepsis—a systemic inflammatory condition that unleashes response through toll-like receptors (TLRs), especially TLR-2 and TLR-4, proteins of a pathway that can be also modulated by physical exercise (De Araújo et al., 2012; Coelho et al., 2013; Hung et al., 2013; Sossdorf et al., 2013; Cavalcante et al., 2017). Whereas the kidney is one of the first organs affected in cases of systemic involvement, acute kidney injury (AKI) is frequently a complication of sepsis (Zarjou and Agarwal, 2011; Abulizi et al., 2017).

Acute kidney injury is a complex clinical disorder and one of the most common and disabling kidney diseases (Venkataraman, 2008; Hoste et al., 2015). The pathophysiology of AKI in sepsis is multifactorial and includes, among other signs, hemodynamic changes, endothelial dysfunction, and inflammatory cell infiltration. It also features acute tubular necrosis due to hypovolemia and consequent low blood perfusion in the tissue. It is associated with severe morbidity and characterized by decreased glomerular filtration function and increased serum urea and creatinine levels (Wan et al., 2008; Zarjou and Agarwal, 2011; Abulizi et al., 2017).

Acute injury is one of the most recurrent diseases in hospitalized patients (Venkataraman, 2008; Hoste et al., 2015) and is also related to increased risk of chronic kidney injury. In experimental models of sepsis, a lipopolysaccharide (LPS) is commonly used, which causes significant damage to multiple organs, resulting in increased inflammatory markers and impaired renal function (Wan et al., 2008; Ahmad et al., 2016; Abulizi et al., 2017).

In the context of AKI, physical exercise has been shown as a tool to improve clinical parameters (Miyagi et al., 2014; Estrela et al., 2017). In 2019, de Lima et al. (2019) showed that preconditioning by aerobic exercises reduces acute ischemic renal injury in rats. In another study, physical exercise has been shown to be able not only to decrease some kidney injury markers but also to diminish cisplatin-induced AKI (Estrela et al., 2017). On the other hand, Sugama et al. (2015) showed that endurance exercises promoted a reduction in renal blood flow leading to ischemia/reperfusion in humans.

Regarding physical exercise, it is worth noting that skeletal muscle contraction releases pro- and anti-inflammatory cytokines during exercise according to stimuli characteristics. Interleukin-6 (IL-6) is the main myokine released by physical exercise and has a pleiotropic effect, being able to promote pro- and anti-inflammatory responses, both locally in the tissue and systemically. The secretion of this cytokine is dependent on the frequency, duration, and intensity of exercise, but studies have pointed to the duration as the most determining factor for the increase of IL-6 (Fischer, 2006; Pedersen, 2017). Moreover, chronic training is able to reduce IL-6 levels both at rest and in response to an acute bout of exercise.

Previous studies have shown that IL-6 is the major pro-inflammatory mediator for AKI and is correlated to the onset and severity of this injury. The main difference between IL-6 response in sepsis and training is that in sepsis, there is a marked increase in circulating levels of tumor necrosis factor alpha (TNF-α) and IL-1β, prior to the increase in IL-6, which does not happen after acute or chronic physical exercise (Pedersen and Febbraio, 2008).

Considering that AKI is a common and disabling syndrome, efforts should be made to minimize the damage caused by this injury. Physical exercise is an important tool to improve the health of patients with kidney diseases, but it is also capable of increasing complications in some conditions. The objective of this work is to evaluate a protocol of chronic physical exercise performed on the treadmill and the effects on LPS-induced AKI.

## Materials and Methods

### Animals

Twelve-week-old male C57BL/6J mice obtained from the animal facilities of the Universidade Federal de São Paulo were used in this study. The animals were fed with diet and water *ad libitum* and housed individually in a temperature-controlled room (22°C), under a 12-h light/dark period. All procedures were conducted under conditions approved by the Animal Ethics Committee [*Comissão de Ética no Uso de Animais* (CEUA)] of the Universidade Federal de São Paulo under number 8686290216. Thirty-one animals initiated the experiment; among these, 28 animals completed the protocol, two mice did not finish the running training, and one mouse did not respond to the LPS dose.

### Exercise Protocol

Before starting the exercise protocol, the animals were familiarized with a motor-driven treadmill by running for 10 min for two consecutive days at incremental speeds (3–9 m/min). After this adaptation period, the animals rested for 48 h and then underwent a maximum speed test (MST) based on a previous publication (Ferreira et al., 2007), which consisted of a 3-min warm-up increasing by 3 m/min until exhaustion as detected by biomechanical alteration. We defined the exhaustion criterion as when the animal touches the back of the treadmill three times in a row, and we did not use electric shock in the test or during the training period.

**FIGURE 1:**
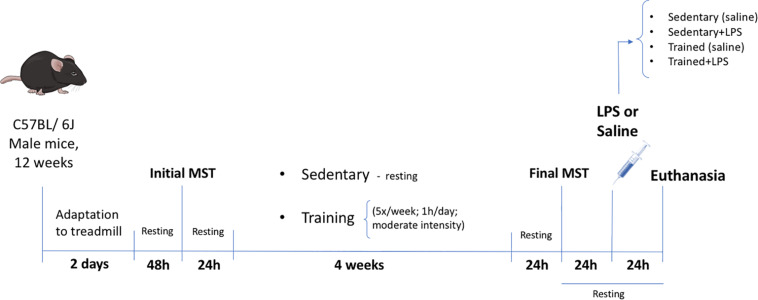
Experimental design. Twelve-week-old male C57BL/6J mice were adapted to a motor-driven treadmill for 2 days (first day 10 min; second day 10 min). After 2 days of resting, the animals were submitted to the first incremental load test until exhaustion (initial MST) for the definition of the training velocity (60% of initial MST). Twenty-four hours after the initial MST, the animals began the treadmill exercise or the resting period for 4 weeks. The last load speed test (final MST) was carried out 24 h after the last exercise session in order to confirm the training effectiveness through the performance improvement. The LPS (5 mg/kg) or saline was administered 24 h after the final MST. All animals were euthanized 24 h after LPS treatment by cervical dislocation.

After this initial MST and considering the homogeneous performance results obtained, the animals were randomly distributed into four groups: (1) sedentary (control) (*N* = 7); (2) sedentary + LPS (*N* = 8); (3) trained (*N* = 8); and (4) trained + LPS (*N* = 8). Animals in the trained groups were submitted to running sessions in a treadmill for 4 weeks (5×/week, 1 h/day) at 60% of the maximum speed according to the initial MST. Two animals did not finish the training protocol and were discarded. The experimental design is presented in Figure 1.

**FIGURE 2:**
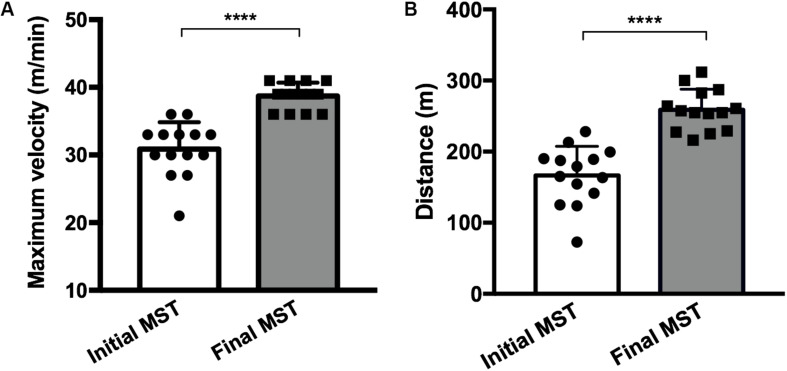
Maximum velocity **(A)** and the total running distance **(B)** achieved before (initial MST) and after (final MST) the exercise protocol showing a performance improvement after training. Fourteen mice in each group (trained, *N* = 7; trained + LPS, *N* = 7; all animals from the trained + LPS group received the LPS injection 24 h after the final MST). Data were compared with *t*-test and Shapiro–Wilk test; ****p < 0.0001. Data are presented as mean ± SD.

Both running in a treadmill and the length of the protocol have been previously published (Sonobe et al., 2015; Fujimaki et al., 2016; Minakaki et al., 2019), and in all the aforementioned studies, the 4-week exercise protocol proved effective. The MST was performed at two time points, 24 h before the first exercise session and 24 h after the last exercise session, in order to evaluate the difference of the performance acquired through maximum velocity and the total running distance achieved before and after the experimental protocol (Figure 2).

#### LPS Treatment

In order to define the LPS dose, we performed a pilot test (data not shown) with three different LPS doses that can be found in the literature (1, 2, and 5 mg/kg). The best result for the presence of nephropathy was found with the 5-mg/kg dose: all animals in this group presented a significant increase in urea and creatinine levels compatible with AKI and zero mortality rate. In addition, the LPS dose used to induce kidney damage has been published before (Huugen et al., 2005; Chen et al., 2018).

The animals were divided into two groups: the first group (LPS+, *N* = 8) received a single dose of LPS 5 mg/kg (*Escherichia coli* O111:B4—L2630—Sigma-Aldrich, EUA) via intraperitoneal (i.p.) injection, and the second group (LPS-, *N* = 7) received saline solution (i.p.). These injections were applied 24 h after the last MST (final MST) and 48 h after the last exercise section (Figure 1). One mouse did not respond to the LPS dose and was discarded; the survival rate after 24 h was 100%.

The animals were euthanized 24 h after the LPS injection, since we aimed to evaluate the responses after an already-established although still acute injury. All animals were euthanized with a combination of xylazine (9.1 mg/kg) and ketamine (91 mg/kg) administered intraperitoneally, and approximately 600 μl of blood was collected by heart puncture. The kidneys were collected and kept frozen at −80°C until the extraction of RNA and protein content.

### RNA Extraction and Real-Time PCR

RNA was extracted with TRIzol (#15596026, Thermo Fisher Scientific) according to the manufacturer’s instructions after processing approximately one half of a kidney in a Precellys (Bertin Instruments). Synthesis of cDNA was conducted with High Capacity cDNA Reverse Transcription kit (Thermo Fisher Scientific, #4368814) using random primers and according to the manufacturer’s instructions. The real-time PCR was performed using 20 ng of cDNA and HOT FIREPol EvaGreen qPCR Mix, ROX (Solis BioDyne, #08-24-00001), 10-μL reaction volume in QuantStudio 3 equipment (Applied Biosystems). Relative quantification was performed with the 2^–ΔΔCt^ method using 18S as the reference gene. The primers used are shown in Table 1. For IL-6 and GAPDH (glycerol-3-phosphate dehydrogenase), we used TaqMan probes (Mm00446190_m1 and Mm99999915_g1).

**TABLE 1:** Base pair sequences of primers used in real-time PCR assays.

Gene	Primers/probes
18S	5′-CCTGCGGCTTAATTTGACTC-3′
	5′-AAGACAAATCGCCTCCACCAAC-3′
PGC1-α	5′-TGC GTG TGT GTA TGT GTG TGT G-3′
	5′-CCT TGT TCG TTC TGT TCA GGT G-3′
TNF-α	5′-GCCTCTTCTCATTCCTGCTTG-3′
	5′-CTGATGAGAGGGAGGCCATT-3′
Bax	5′-CGGCGAATTGGAGATGAACTG-3′
	5′-GCAAAGTAGAAGAGGGCAACC-3′
Bcl-2	5′-ACCGTCGTGACTTCGCAGAG-3′
	5′-GGTGTGCAGATGCCGGTTCA-3′
NGAL	5′-ATGTGCAAGTGGCCACCACG-3′
	5′-CGCATCCCAGTCAGCCACAC-3′
Kim-1	5′-TGTCGAGTGGAGATTCCTGGATGGT-3′
	5′-GGTCTTCCTGTAGCTGTGGGCC-3′
TLR-2	5′-TTTCACCACTGCCCGTAGAT-3′
	5′-ATGTAACGCAACAGCTTCAGGA-3′
TLR-4	5′-TGTTCTTCTCCTGCCTGACA-3′
	5′-TGTCATCAGGGACTTTGCTG-3′
MyD88	5′-GAGTGGAGCGTGGCAGTAAA-3′
	5′-AAGGTTTAAGAGAGGTGACTGGC-3′
ALPL	5′-CCTGACTGACCCTTCGCTCT-3′
	5′-CTGCTTGGCCTTACCCTCAT-3′
AOAH	5′-ATGAAGGCTGATGTGGTGTG-3′
	5′-AGGACCTCCTGAGGACTTGT-3′

*18S, 18S ribosomal RNA; PGC1-α, peroxisome proliferator-activated receptor-gamma coactivator 1α; TNF-α, tumor necrosis factor alpha; BAX, Bcl-2-associated X; BCL-2, B-cell lymphoma 2; NGAL, neutrophil gelatinase-associated lipocalin; Kim-1, kidney injury molecule 1; TLR-2, toll-like receptor 2; TLR-4 toll-like receptor 4; MyD88, myeloid differentiation primary response 88; ALPL, alkaline phosphatase; AOAH, acyloxyacyl hydrolase.*

### Protein Extraction and Western Blot

Kidney tissue was homogenized in extraction buffer containing phosphatases and protease inhibitors 100 mM Tris-HCl (pH 7.5), 1% Triton X-100, 10% sodium dodecyl sulfate (SDS), 10 mM EDTA, 100 mM sodium fluoride, 10 mM sodium pyrophosphate, 10 mM sodium orthovanadate, 2 mM phenylmethylsulfonyl fluoride (PMSF), and 0.1 mg aprotinin/ml at 14,000 rpm for 40 min at 4°C. Protein concentrations were determined by the Bradford assay (Bio-Rad Laboratories, Hercules, CA, United States). Extracts were used for Western Blot (WB) analysis.

Briefly, the proteins were transferred to nitrocellulose membranes in Tris-glycine buffer (Bio-Rad). The membranes were incubated overnight with 5% blotting-grade blocker containing 5% BSA in Tris-buffered saline, plus 0.1% Tween 20 solution and specific antibodies. The primary antibodies anti-TLR-4 antibody (1:1,000, Santa Cruz, #293072) or anti-myeloid differentiation primary response 88 (MyD88) (1:10,000, Santa Cruz, #11356) were included. IgG anti-rabbit, HRP-conjugated antibody (1:20,000, #A6154 Sigma-Aldrich) was used as a secondary antibody for all blotting assays. The blots for all proteins of interest were visualized using the substrate SuperSignal West Pico (Pierce). All proteins of interest were analyzed by densitometry. The relative expression levels were normalized by dividing the values for the protein of interest by the total protein.

The loading control in WB assays was performed by normalizing the WB bands by the total protein, stained on the membrane by the stain-free method (Bio-Rad) (Taylor et al., 2013). The band intensities were normalized by the respective total lane protein intensity, using the Image Lab software v.6.0.0 (Bio-Rad).

### Renal Function

Serum urea and creatinine levels were used to determine renal function. Blood was collected by heart puncture. All samples were analyzed with a colorimetric assay, using the commercial kit Labtest, Lagoa Santa, Brazil.

### Enzyme-Linked Immunosorbent Assay

Serum levels of IL-6 were determined using a DuoSet Mouse Enzyme-Linked Immunosorbent Assay (ELISA) kit (no. DY406, lot 331611, R&D Systems, MN, United States) according to the manufacturer’s instructions.

### Enzymatic Activity (NAG and MPO) and GSH Levels

In order to analyze the *n*-acetyl-β-D-glycosaminidase (NAG) activity, the kidney was homogenized and centrifuged, and the supernatant was incubated with 100 μl of *p*-nitrophenyl-*n*-acetyl-β-D-glucosaminide (Sigma-Aldrich Co., LLC). The reaction was stopped by adding 100 μl of 0.2 M glycine buffer (pH 10.6), and the hydrolysis of the substrate was determined by measuring absorption at 400 nm.

For the myeloperoxidase (MPO) activity, the kidney was homogenized in pH 4.7 buffer and centrifuged. The pellets were submitted to freeze–thaw cycles, and the MPO activity was assayed by measuring the change in absorbance [optical density (OD)] at 450 nm using tetramethylbenzidine (1.6 mM) and H_2_O_2_ (0.3 mM). The reaction was stopped by adding 50 ml of H_2_SO_4_ (4 M).

To measure the reduced glutathione (GSH), the kidney was homogenized in phosphate buffer (pH 6.5, 0.1 M). The resulting supernatant was mixed with 12.5% trichloroacetic acid (TCA), incubated, and centrifuged, as previously described (Coutinho et al., 2016; de Araújo et al., 2018). Glutathione was assayed by measuring the change in absorbance (OD) at 415 nm after pipetting 5,5′-dithio-bis(2-nitrobenzoic acid) (DTNB). The results of this assay are expressed as nanomolars per milligram.

### Histological Analysis

The kidney was fixed in 4% paraformaldehyde for 24–48 h at room temperature. The paraffin-embedded kidney was sectioned and stained with hematoxylin and eosin (H&E) to visualize morphology. Optic light microscopy was employed to analyze the samples. Images were acquired at 40× magnification. The injury score was determined based on the percentage of tubules showing luminal casts, cell detachment, or dilation and assigned according to the following scale: 0 = 0–5%, I = 6–25%, II = 26–50%, III = 51–75%, and IV > 75%. Histological analyses were blindly performed by a kidney pathologist.

### Statistical Analysis

GraphPad Prism v8.2.1 was used for statistical analysis. Data are shown as mean ± SD. Data were compared by two-way ANOVA followed by Tukey multiple comparisons test and unpaired, non-parametric Mann–Whitney test. Statistical significance was considered for *p* < 0.05 (GraphPad Software, San Diego, California, United States)^1^.

## Results

### Physical Exercise Proves to Be Effective Through the Increase of Maximum Velocity and the Total Running Distance Achieved After the Experimental Running Protocol

The MST performed 4 weeks after training showed an increase in maximum velocity (*p* < 0.0001) and total running distance (*p* < 0.0001) in all trained animals (Figures 2A,B), demonstrating that the protocol model was effective.

In order to analyze if our protocol was able to promote alterations in the kidney tissue and considering that several physiological adaptations of physical exercise are mediated by peroxisome proliferator-activated receptor-gamma coactivator 1α (PGC1-α; Handschin and Spiegelman, 2008), we checked the mRNA of this cofactor and found that our training protocol increased PGC1-α levels (Supplementary Figure 1) in the kidney, suggesting a positive effect of training in this tissue.

### LPS Treatment Impairs Renal Function and Physical Exercise Exacerbates Kidney Damage

**FIGURE 3:**
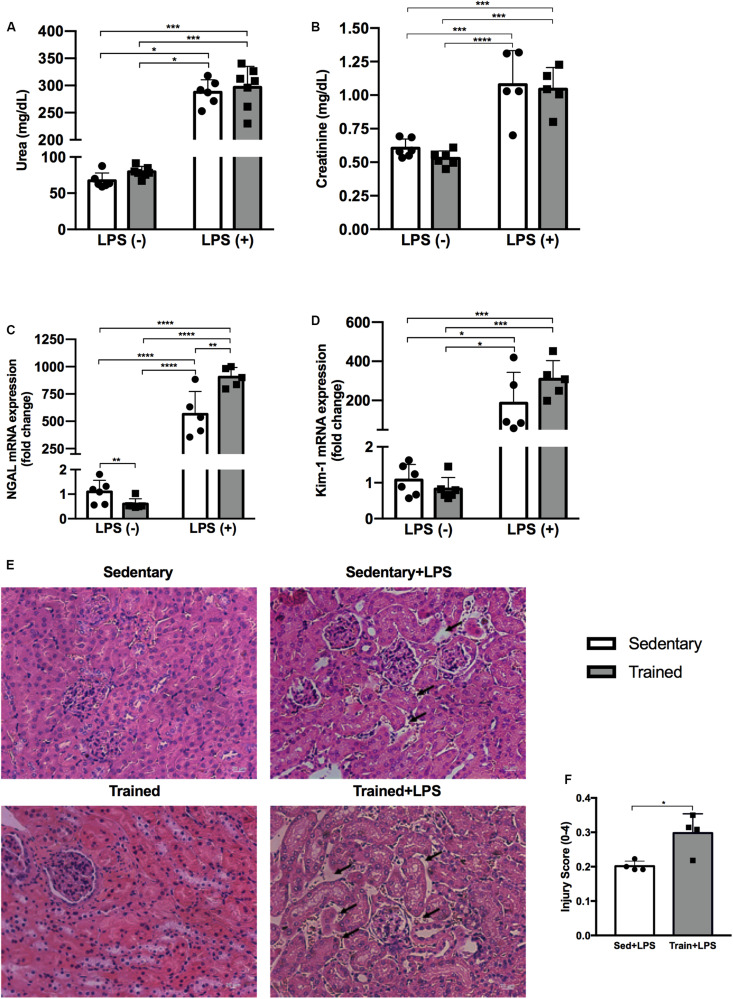
Serum urea **(A)** and creatinine **(B)** concentrations. Real-time PCR of NGAL **(C)** and Kim-1 **(D)** in the kidney. Histological images of the kidney with black arrows indicating an expansion of the tubulointerstitial space **(E)**. Injury score of histological analysis **(F)**. Animals treated with a single injection of LPS (5 mg/kg) or saline. Data were compared by two-way ANOVA with Tukey multiple comparisons test **(A–D)**. Injury score between sedentary + LPS and trained + LPS compared with *t*-test and Shapiro–Wilk test; *p < 0.05, **p < 0.01, ***p < 0.001, ****p < 0.0001. Interaction between LPS and exercise was found in NGAL mRNA analysis *p* = 0.0016. Data are presented as mean ± SD. Five to seven mice in each group.

The administration of LPS caused an increase in urea concentration in the sedentary + LPS group (*p* < 0.0001) and in the trained + LPS group (*p* < 0.0001). The creatinine levels were also increased in the sedentary + LPS (*p* < 0.001) and trained + LPS (*p* = 0.001) animals, demonstrating that the dose applied was able to compromise renal function with no differences observed between sedentary and trained animals (Figures 3A,B). In order to analyze whether LPS modulates kidney injury-related gene expression and if the training protocol could interfere in these responses, we checked neutrophil gelatinase-associated lipocalin (NGAL) and kidney injury molecule 1 (Kim-1), and both gene expressions were higher when animals received LPS (Figures 3C,D). Regarding NGAL, the increase was significant in trained + LPS (*p* < 0.01) compared to sedentary + LPS animals. Histological analyses revealed a low injury score, but the animals from the trained + LPS group showed a clear expansion of the tubulointerstitial space (*p* < 0.05) as shown in Figures 3E,F.

### LPS Treatment Increased mRNA Expression of Pro-inflammatory Cytokines in the Kidney Tissue. Physical Exercise Did Not Prevent This Response

**FIGURE 4:**
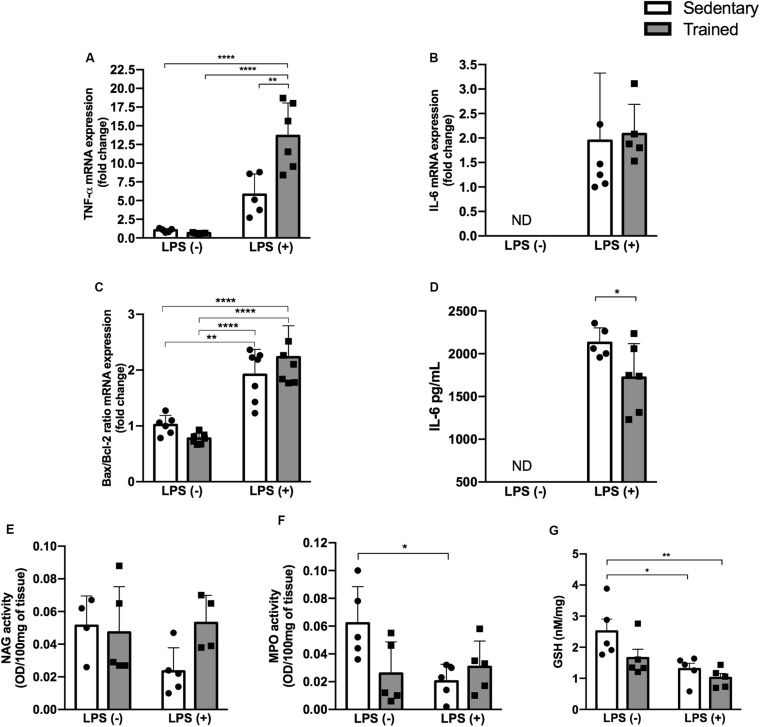
Enzymatic activity and GSH and IL-6 levels and gene expression. Real-time PCR of TNF-α **(A)**, IL-6 **(B)**, and Bax/Bcl-2 ratio **(C)** in the kidney. Serum IL-6 concentration **(D)**. Enzymatic activity of NAG **(E)**, MPO **(F)**, and GSH levels **(G)** in the kidney of mice treated with a single injection of LPS (5 mg/kg) or saline. Data were compared by two-way ANOVA with Tukey multiple comparisons test **(A,C,E,F,G)** or unpaired, non-parametric Mann–Whitney test **(B,D)** between LPS groups; *p < 0.05, **p < 0.01, ***p < 0.001, ****p < 0.0001. Interaction between LPS and exercise was found in TNF-α mRNA (*p* = 0.0033) and MPO activity (*p* = 0.0232) analysis. Data are presented as mean ± SD. Five to seven mice in each group.

Pro-inflammatory cytokines TNF-α and IL-6, as well as the Bax/Bcl-2 ratio, were higher in the groups receiving LPS (Figures 4A–C). Physical exercise prior to LPS administration raised the TNF-α expression (*p* < 0.001) compared to the sedentary + LPS group, as presented in Figure 4A.

### LPS Administration Modulated the Activity of Enzymes Linked to Oxidative Stress With a Slight Difference in Sedentary + LPS and Trained + LPS Groups Compared to Control

In order to investigate whether the relationship between physical exercise and the LPS pathway could be linked to oxidative stress, we checked NAG and MPO activities and GSH levels (Figures 4E–G). We observed a decrease in MPO activity in the sedentary + LPS (*p* < 0.05) group and a decrease in GSH levels in the sedentary + LPS (*p* < 0.05) and trained + LPS (*p* < 0.01) groups, suggesting that LPS promoted oxidative stress in kidney tissue. This effect was even higher in trained + LPS animals compared to control (Figure 4G).

### Physical Exercise Modulates the Expression of TLR Pathway Genes in mRNA and Protein Levels

**FIGURE 5:**
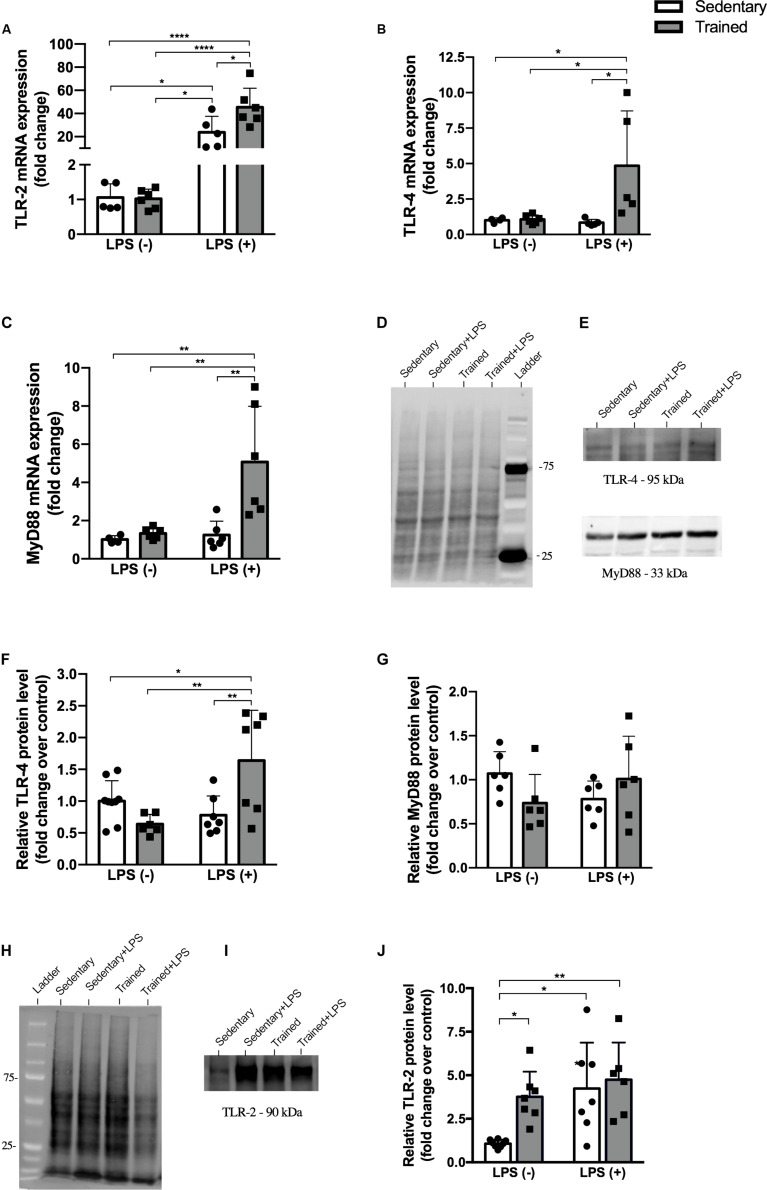
Protein and gene expression. Real-time PCR of TLR-2 **(A)**, TLR-4 **(B)**, and MyD88 **(C)**. Western blot for TLR-4, MyD88, and TLR-2 **(D–J)** in the kidney of mice treated with a single injection of LPS (5 mg/kg) or saline. Total proteins stained on the membrane were used for TLR-4 and MyD88 **(D)** and TLR-2 **(H)** normalization. Data were compared by two-way ANOVA with Tukey multiple comparisons test; *p < 0.05, **p < 0.01, ***p < 0.001, ****p < 0.0001. Interaction between LPS and exercise was found in TLR-2 mRNA (*p* = 0.0315), TLR-4 mRNA (*p* = 0.0388), and TLR-4 protein levels (*p* = 0.0001) analysis. Data are presented as mean ± SD. Five to seven mice in each group.

In order to establish the role of physical exercise in the modulation of LPS pathway when associated with the endotoxin, we performed gene and protein experiments and observed an increased expression of TLR-2, TLR-4, and MyD88 in mRNA levels (Figures 5A–C). No difference was observed for the protein expression of MyD88, but the expression of TLR-4 was increased in LPS groups and even higher in the trained + LPS group compared to the sedentary + LPS group at mRNA (*p* < 0.05) and protein (*p* < 0.01) levels (Figures 5D–G). Compared to those in the control, the TLR-2 protein levels were increased in all groups: trained (*p* < 0.05), sedentary + LPS (*p* < 0.05), and trained + LPS (0 < 0.01) groups as shown in Figures 5H–J.

### Physical Exercise Modulates the Expression of a Hepatic Enzyme That Is Part of LPS Detoxification

**FIGURE 6:**
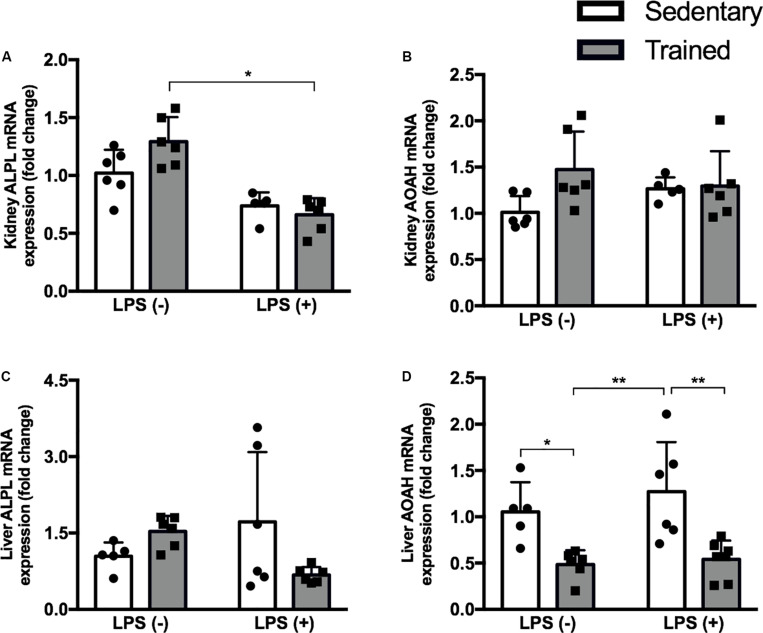
Real-time PCR of ALPL **(A)** and AOAH **(B)** in the kidney and ALPL **(C)**, and AOAH **(D)** in the liver of mice treated with a single injection of LPS (5 mg/kg) or saline. Data were compared by two-way ANOVA with Tukey multiple comparisons test; *p < 0.05, **p < 0.01, ***p < 0.001, ****p < 0.0001. Interaction between LPS and exercise was found in kidney ALPL mRNA (*p* = 0.0272) and liver ALPL mRNA (*p* = 0.0222) analysis. Data are presented as mean ± SD. Five to seven mice in each group.

In order to investigate whether our exercise protocol could interfere in the gene expression of enzymes related to LPS detoxification, we checked the expression of alkaline phosphatase (ALP) and acyloxyacyl hydrolase (AOAH) in the kidney and liver (Figures 6A–D). We observed a decrease of AOAH in the liver, the major organ related to LPS detoxification, in both trained groups with and without LPS.

## Discussion

Physical exercise is known not only for its ability to promote metabolic adaptations that improve health but also for attenuating harmful effects in situations of injury or illness in several organs and systems (Hung et al., 2013; El-Kader et al., 2014; Wegner et al., 2014; Sharman et al., 2015). Over the years, an active lifestyle and regular supervised exercises have been recommended to people of all ages and in a number of syndromes and pathologies (Kelley et al., 2015; Catania et al., 2017; Mora and Valencia, 2018).

Acute kidney injury has an occurrence of 30–40% in intensive care unit patients (Hoste et al., 2015), and one of its main causes is the endotoxemia promoted by LPS, a naturally found component present in the outer membrane of most gram-negative bacteria, which can trigger cytokine secretion and a systemic inflammatory process (Chen et al., 2018). Organ impairment by our AKI model was confirmed by increased blood urea and creatinine levels, as well as increased gene expression of well-described AKI markers: NGAL and Kim-1 (Mishra et al., 2004; Han et al., 2018). It is worth noting that only NGAL showed a significant difference between sedentary + LPS and trained + LPS groups; and the increase of urea and Kim-1 in trained + LPS was higher than that observed for sedentary + LPS animals, when compared to control.

Increased injury markers observed in this study corroborate the literature data and have been previously reported (Mishra et al., 2004; Humphreys et al., 2013; Tan et al., 2016). Even at different LPS doses and moments of euthanasia, urea and creatinine levels and NGAL and/or Kim-1 were elevated (Han et al., 2018; Wang et al., 2018; Plotnikov et al., 2019). Regarding exercise, it is important to take variations in the protocols into account when analyzing the results. In strenuous protocols that promote tissue damage secondary to exercise, AKI markers are frequently increased (Mansour et al., 2017), but this is not reported in moderate-intensity protocols.

Histopathological analysis revealed initial alteration mainly in trained + LPS animals. This is in line with the literature, given that sepsis-induced AKI is usually associated with a lower rate of tubular apoptosis and necrosis when compared to other forms of AKI (Dellepiane et al., 2016). Alternatively, the absence of tissue damage could be explained by the dose of LPS and the time of euthanasia. Islam et al. (2019) reported some extent of tissue injury, but in that case, the mice had received a dose of 8 mg/kg of LPS. In a study in which C57BL/6 mice were given LPS, Zhang et al. (2018) reported “swollen tubular epithelial cells and indistinct brush border” 24 h after LPS injection, but the dose was twice as high as the dose used in this study.

In our model, tissue damage was possibly at an early stage, but trained + LPS animals showed morphological abnormalities, such as a clear expansion of the tubulointerstitial space. These data, contrary to our initial hypothesis, shows that the training protocol was not able to protect the kidney from LPS-induced injury, but it appears to worsen the condition.

Tumor necrosis factor alpha gene expression was also increased in LPS groups, mainly in the trained + LPS group. An elevated Bax/Bcl-2 ratio suggests the presence of apoptosis, and the increase of TNF-α and IL-6 confirms the inflammatory pattern present in AKI (Schrier, 2010; Miyagi et al., 2014; Estrela et al., 2017). Among these genes, the increase of TNF-α gene expression in the trained + LPS group could be due to some interaction between training and the signaling cascade triggered by LPS.

With respect to sepsis and endotoxemia models, both are known to be associated with the generation of reactive oxygen species (ROS) and oxidant injury, responses that are normally present in AKI (Chen et al., 2018; Gu et al., 2019). We measured the activity of enzymes related to oxidative stress and the GSH levels, and we found a decrease in reduced GSH in both LPS groups. These data are in accordance with the literature (Islam et al., 2019; Zhang et al., 2019). Nonetheless, the trained + LPS group showed a marked reduction of GSH compared to control, but with no differences between sedentary + LPS and trained + LPS groups.

Its already known that reduced GSH is one factor that leads to increased LPS-induced oxidative stress (Chen et al., 2018). Physical exercise has beneficial effects on oxidative stress, but this relation can be complex, since it is dependent on the intensity, mode, and frequency of exercise. During exercise, a transient state of oxidative stress occurs, but these alterations promote adaptations to the stimuli (Pingitore et al., 2015; Korsager Larsen and Matchkov, 2016). Based on this, we hypothesized that physical exercise prior to LPS-induced AKI could decrease the oxidative stress through the maintenance of GSH levels. However, when compared to control, these levels were even lower. Therefore, we can conclude that there was a reduction in GSH levels in the presence of LPS, and our moderate-intensity protocol was not able to reverse it.

Since physical exercise seems to interfere in LPS action, we looked to the receptors of this molecule. LPS acts through the interaction of its lipid A portion, which is responsible for the LPS endotoxic activity, activating TLR-2 and TLR-4 cascades and promoting NF-κB translocation to the nucleus and the expression of pro-inflammatory cytokines (Park and Lee, 2013). In our model, TLR-2 and TLR-4 gene expressions were increased in the trained + LPS group, and the protein levels of TLR-4, the main LPS receptor, were also increased in the trained + LPS animals. Interestingly, when we look at TLR-2, both exercise and LPS were able to modulate this receptor. Our moderate-intensity protocol upregulated TLR-2 in protein levels, and the increase of this receptor by exercise has been shown before (Zheng et al., 2015). The overexpression of TLR-2 could have contributed to the increase of damage when associated with LPS as we observed in other parameters.

The mechanisms involved in downregulation or upregulation of TLRs are far from being fully understood. Authors have shown the involvement of transcription factors, miRNAs, cytokines, and proteins (Sadeghi et al., 2016; Liu et al., 2017), but so far, it is not clear how a highly endotoxic molecule receptor is upregulated in the presence of LPS. Both TLR-2 and TLR-4 receptors can activate the MyD88 pathway, which involves the early activation of NF-κB. However, TLR-4 can activate the MyD88-independent pathway, which involves TRIF, IFN, IP-10, and the late phase of NF-κB activation (Kawai et al., 2001; Mandal et al., 2010). Our results show an increased MyD88 gene expression; however, MyD88 protein levels were not increased in the trained + LPS group when compared to the sedentary + LPS group.

As described before, the major source of LPS is Gram-negative bacteria that are part of intestinal microbiota. Once released by cellular division or death, there are several mechanisms to limit the entrance of LPS into the bloodstream. The main organ related to LPS detoxification is the liver, through enzymes expressed on hepatocytes (ALP) and Kupffer cells (AOAH). Alkaline phosphatase cleaves the phosphate groups of lipid A, leading to a non-toxic molecule, while AOAH promotes LPS deacylation, thus reducing the activity and practically inactivating LPS (Guerville and Boudry, 2016).

In the literature, ALP modulation has been previously linked to physical exercise, but only in a few studies and with different results according to the exercise protocol (Lin et al., 2015; Chen et al., 2016; Traiperm et al., 2016). Regarding AOAH, no study investigating the modulation of this enzyme by physical exercise was found. Our hypothesis is that our protocol could promote a reduction in ALP or AOAH enzyme and, consequently, allow a greater amount of circulating active LPS capable of binding to TLR-4 and promoting the differences we found between the sedentary + LPS and trained + LPS groups.

We analyzed the gene expression of ALP and AOAH in the kidney and in the liver of mice, and we found that physical exercise reduced hepatic AOAH mRNA expression in both trained groups, with and without LPS. Both the phosphate group and the acyl chains of LPS molecule are important for TLR-4 signaling, but adequate binding is dependent mainly on the acyl chains, which is exactly the portion where AOAH acts (Park and Lee, 2013). For the first time, this study shows that an exercise protocol is capable of modulating AOAH to some extent.

Given the above, we can conclude that our exercise protocol accentuated LPS-induced AKI, increasing the expression of TLR-4. The positive regulation of TNF-α, NGAL, and TLR-4 observed in trained + LPS compared to sedentary + LPS animals may be due to the decreased AOAH in the liver, the main LPS detoxification enzyme. Considering this decrease in hepatic AOAH, the amount of active LPS in the bloodstream may be higher in trained animals submitted to LPS injection. Further investigation is required to confirm this suggestion and to investigate the impact of different exercise protocols on liver enzymes involved in LPS clearance.

## Data Availability Statement

All datasets generated for this study are included in the article/Supplementary Material.

## Ethics Statement

The animal study was reviewed and approved by Federal University of São Paulo – Animal Ethics Committee (CEUA) under the number 8686290216.

## Author Contributions

TH conceived the study, designed and drafted the work, collected the sample, performed the experiments, analyzed and interpreted the data, and wrote the manuscript. GE performed the real-time qPCR experiments, interpreted the data, and reviewed the manuscript. LF-L performed the enzymatic assay experiment, interpreted the data, and reviewed the manuscript. MP and MG performed the animal training, collected the sample, and reviewed the manuscript. TA-S performed the Western blot experiments, collected the sample, and reviewed the manuscript. AA performed the LPS injection and collected the sample. JB-C performed the histological analyses. RA conceived the study, designed and drafted the work, analyzed and interpreted the data, supervised the study, reviewed the manuscript, and acquired funding. All authors contributed to the article and approved the submitted version.

## Conflict of Interest

The authors declare that the research was conducted in the absence of any commercial or financial relationships that could be construed as a potential conflict of interest.

## Footnotes

1 www.graphpad.com
