# Supplementary material for: Physical Exercise Exacerbates Acute Kidney Injury Induced by LPS via Toll-Like Receptor 4
Source: Front Physiol. 2020 Jul 17;11:768. doi: 10.3389/fphys.2020.00768 (PMC7380174; doi:10.3389/fphys.2020.00768)
Supplement: Supplementary file 1 [file Data_Sheet_1.PDF]

Supplementary Material

1 Supplementary Data

1.1. Exercise training and PGC1-α mRNA in kidney tissue

In order to elucidate if our exercise protocol has promoted some alterations in the kidney tissue, we checked the kidney mRNA of this cofactor and found that our training protocol increased PGC1-α mRNA levels (supplementary figure 1), suggesting a positive effect in this tissue. However, it failed to protect the kidney in our LPS-induced AKI.

1.2. Daily food intake

In order to analyze if the exercise protocol prior to LPS injection could interfere in the food intake, we measure it from 3 days before the LPS or saline injection to 24h after the procedure (supplementary figure 2). The exercise training did not modulate the food intake.

1.3. Animals and kidney mass weight

On the day of euthanasia, all animals were weighed (supplementary figure 3A). After the procedure, the kidneys were extracted and also weighed (supplementary figure 3B). Both, the training and que LPS injection did not modulate the result.

2 Supplementary Figures and Tables

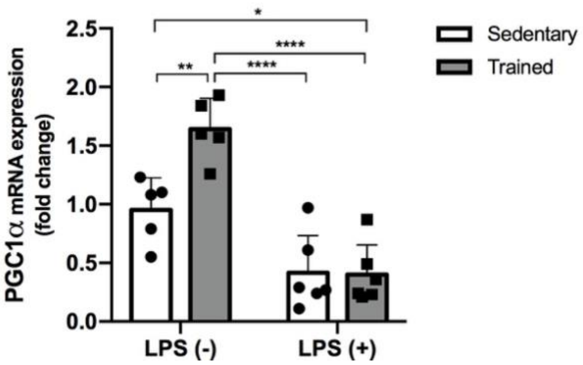

Supplementary figure 1. Real-Time PCR of PGC1-α in the kidney of mice treated with a single injection of LPS (5mg/Kg) or saline. Data were compared by Two-Way ANOVA with Tukey multiple comparisons test; *p<0.05, **p <0.01, ***p <0.001, ****p<0.0001. Data are presented as Mean ± SD. Five to seven mice in each group.

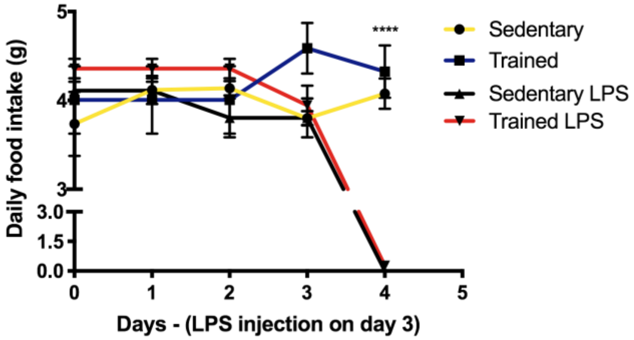

Supplementary Figure 2. Daily food intake. The exercise protocol prior to LPS injection did not interfere in the food intake of animals (mice treated with a single injection of LPS (5mg/Kg) or saline). 24h after the administration of endotoxin, both groups that receive LPS decrease the food intake compared to groups that received a saline solution. Data were compared by Two-Way ANOVA with Tukey multiple comparisons test; ****p<0.0001. Data are presented as Mean ± SD. Seven mice in each group.

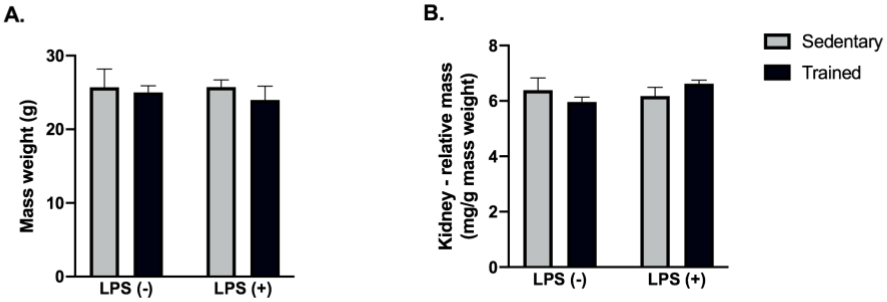

Supplementary Figure 3. Animal mass weight (**A**); Kidney relative mass weight (**B**). Data were compared by Two-Way ANOVA with Tukey multiple comparisons test. Data are presented as Mean ± SD. Seven mice in each group.
